# Serogroups of *Dichelobacter nodosus,* the cause of footrot in sheep, are randomly distributed across England

**DOI:** 10.1038/s41598-020-73750-5

**Published:** 2020-10-08

**Authors:** Naomi S. Prosser, Emma M. Monaghan, Laura E. Green, Kevin J. Purdy

**Affiliations:** 1 School of Life Sciences, The University of Warwick, Gibbet Hill Campus, Coventry, CV4 7AL UK; 2 Institute of Microbiology and Infection, College of Life and Environmental Sciences, University of Birmingham, Edgbaston, Birmingham, B15 2TT UK; 3 Present Address: School of Veterinary Medicine and Science, University of Nottingham, Sutton Bonington Campus, Leicestershire, LE12 5RD UK

**Keywords:** Infectious-disease epidemiology, Microbial communities, Pathogens, Infectious diseases

## Abstract

We present the largest and most representative study of the serological diversity of *Dichelobacter nodosus* in England. *D. nodosus* causes footrot and is one of the top five globally important diseases of sheep. The commercial vaccine, containing nine serogroups, has low efficacy compared with bivalent vaccines. Our aim was to investigate the prevalence and distribution of serogroups of *D. nodosus* in England to elucidate whether a bivalent vaccine could protect the national flock. Farmers from 164 flocks submitted eight interdigital swabs from eight, preferably diseased, sheep. All serogroups, A–I, were detected by PCR in 687/1150 *D. nodosus* positive swabs, with a prevalence of 2.6–69.3% of positive swabs per serogroup. There was a median of two serogroups per flock (range 0–6). Serogroups were randomly distributed between, but clustered within, flocks, with 50 combinations of serogroups across flocks. H and B were the most prevalent serogroups, present in > 60% of flocks separately but in only 27% flocks together. Consequently, a bivalent vaccine targeting these two serogroups would protect 27% of flocks fully (if only H and B present) and partially, if more serogroups were present in the flock. We conclude that one bivalent vaccine would not protect the national flock against footrot and, with 50 combinations of serogroups in flocks, flock-specific vaccines are necessary.

## Introduction

*Dichelobacter nodosus* is a gram negative, anaerobic bacterium and the causal agent of footrot^1–3^, the principal cause of lameness in sheep in the UK^4^. Footrot presents as an interdigital dermatitis which sometimes progresses to severe footrot with destruction of the epidermis resulting in separation of the hoof horn from the underlying tissue^5^. Transmission of *D. nodosus* occurs between sheep via pasture^6^. The survival of *D. nodosus* in soil is influenced by soil type, temperature and moisture^6^. Footrot is endemic in the UK and is present in over 90% of flocks in England, with a mean prevalence of 4.5% and 3.1% for interdigital dermatitis and severe footrot lesions respectively, accounting for 68% of foot lesions^4^. Footrot is estimated to cost the UK sheep industry about £80 million per annum in treatments and lost production^7^ and is of considerable concern to sheep farmers^8,9^.

*D. nodosus* is subdivided into ten serogroups (A–I and M) which differ in their fimbrial antigens^10^. All ten serogroups have been detected in the UK^11,12^. To date the prevalence of serogroups has been reported in studies with only 34–58 non-randomly selected flocks per study and serogroups H and B have dominated^11–14^. It is common for several serogroups of *D. nodosus* to be detected concurrently in flocks and on feet in the UK, with a median of two and maximum of four serogroups per flock, with > 1 serogroup detected from 10% of sheep and 60% of flocks using culture methods^12–14^. Multiple serogroups are also detected on feet and in flocks in other countries, e.g. McPherson et al^15^. Previous UK studies have used culture and slide agglutination to detect serogroups, but PCR is more sensitive^15^ consequently the number of serogroups per flock is likely to be higher than previous estimates.

A vaccine that provides effective protection against footrot is highly desirable because it would prevent sheep from becoming lame and so improve welfare and productivity^7^. It would also reduce the need for treatment with antibiotics, which would significantly reduce their use in sheep farming because lameness accounts for over 60% of antibiotic use in sheep^16^. Despite the poor natural immune response of sheep to footrot, vaccination does initiate immunity^17^ to homologous serogroups^18,19^. Individual sheep differ in their response to the vaccine, both in the total amount of antibody produced and the relative proportion of antibodies against each serogroup in the vaccine^18,20^. Serogroup antigens also differ in their immunodominance^20,21^. Because several serogroups are often present in flocks, researchers have tested the efficacy of bivalent vaccines that target the specific serogroups in a flock^22^. Sequential administration of tailored mono- and bivalent vaccination over time has had some success in eliminating severe footrot from flocks in Australia^23^. However, as the number of serogroups present in a flock increases, increased testing and vaccination effort is required to eliminate severe footrot^23^. Multivalent vaccines against *D. nodosus* confer lower immunity per strain compared with monovalent vaccines because of antigenic competition^24^. Sheep vaccinated with multivalent vaccines, compared with monovalent vaccines, have significantly lower antibody titres and these decline more rapidly, therefore the sheep has both less protection and for a shorter duration^24^. Alternatives to a fimbrial vaccine that provide cross-protection between serogroups would be advantageous, but these have not been marketed, and research is ongoing in this area^25,26^. The commercial multivalent fimbrial vaccine, Footvax, is protective for up to 5 months^27^ and field studies indicate only partial protection^28,29^. In a recent observational study of 1260 flocks in England, Footvax reduced the proportional prevalence of lameness by an average of 20%, resulting in an absolute mean reduction in lameness of 1%^4^, indicating that vaccination might not be cost effective. Consequently, a bivalent vaccine could be of major benefit to the control of footrot in sheep in the UK if it protected a large proportion of the national flock for longer than six months with a higher magnitude of protection than Footvax.

The aims of the study were to investigate the national distribution, geographical co-location and flock prevalence of nine serogroups (A–I) of *D. nodosus* in England and to test the hypotheses that one bivalent vaccine could be used across the national flock, or several could be used with regional variation in the selected serogroups. We also hypothesised that serogroups in flocks were influenced by use of Footvax or biosecurity practices. Farmers were recruited from 722 compliant respondents to a previous study who were originally from a random sample of 4000 farmers^4^. The 164 /722 farmers who participated in the current study were representative of the original random sample^30^. Participants were asked to complete a questionnaire^30^ and send eight swabs from eight, preferably footrot-affected, feet of eight sheep.

## Results

### Descriptive summary of the dataset

**Figure 1:**
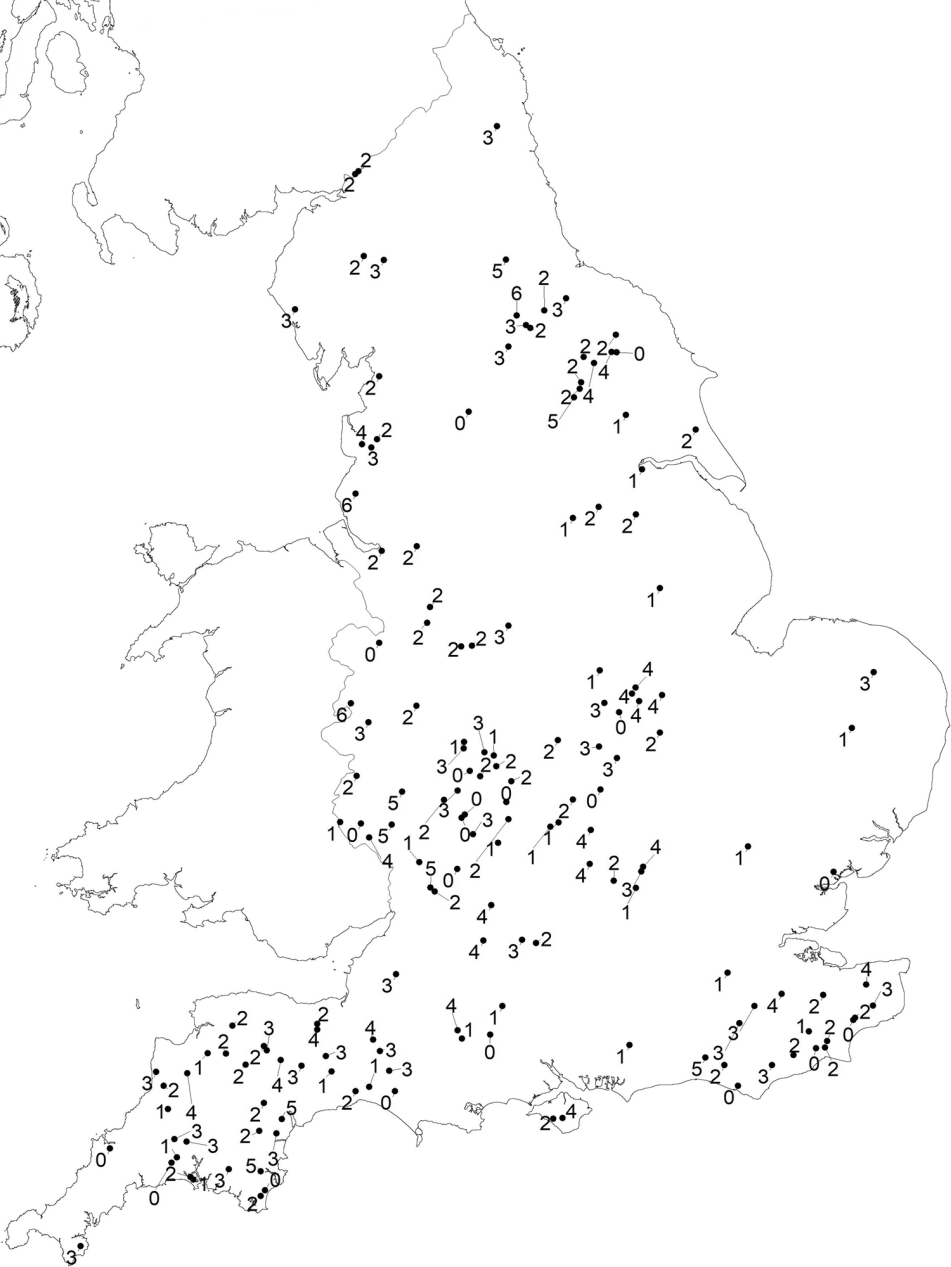
The geographical location of 164 flocks with the number of serogroups of *D. nodosus* detected per flock (range 0–6). The map was created in R statistical software (version 3.5.1)^48^.

In total 1150 swabs from 164 flocks throughout England (Dataset I; Fig. 1) were tested for load and serogroup of *D. nodosus* (3450 qPCR tests and 6183 PCR tests). There were 156 (95.1%) flocks with at least one sample from a footrot-affected foot and 153 (93.3%) with at least one *D. nodosus* positive swab (Dataset II). Flock size and prevalence of lameness and severe footrot were not associated with the number of *D. nodosus* positive swabs submitted (Supplementary Table 1). There were 138 (84%) flocks with 566 swabs from footrot-affected feet and 687 (59.7%) swabs positive for *D. nodosus.* All 687 *D. nodosus* positive swabs were positive for *aprV2* and 74 swabs were also positive for *aprB2.* The median usable number of swabs per flock was 7 (range 3–8) with a median of 4 (range 0–8) *D. nodosus* positive swabs per flock (Table 1).

**Table 1:** Number of swabs per flock by lesion type and *D. nodosus* detection from 164 flocks.

	Median	Range
Number of swabs submitted	8	3–8
Number of feet with Footrot	7	0–8
Number of healthy feet	0	0–7
Number of other lesions status of feet	0	0–8
Number of unknown lesion status of feet	0	0–8
Number of usable swabs	7	0–8
Number of *D. nodosus* positive swabs	4	0–8
Number of *D. nodosus* positive, serogroup negative swabs	1	0–7

Footrot = severe footrot or interdigital dermatitis lesion, Healthy = no lesion, Other = one or more lesions that did not include footrot, Unknown = lesion status not known.

### Number of serogroups by feet and flock

**Figure 2:**
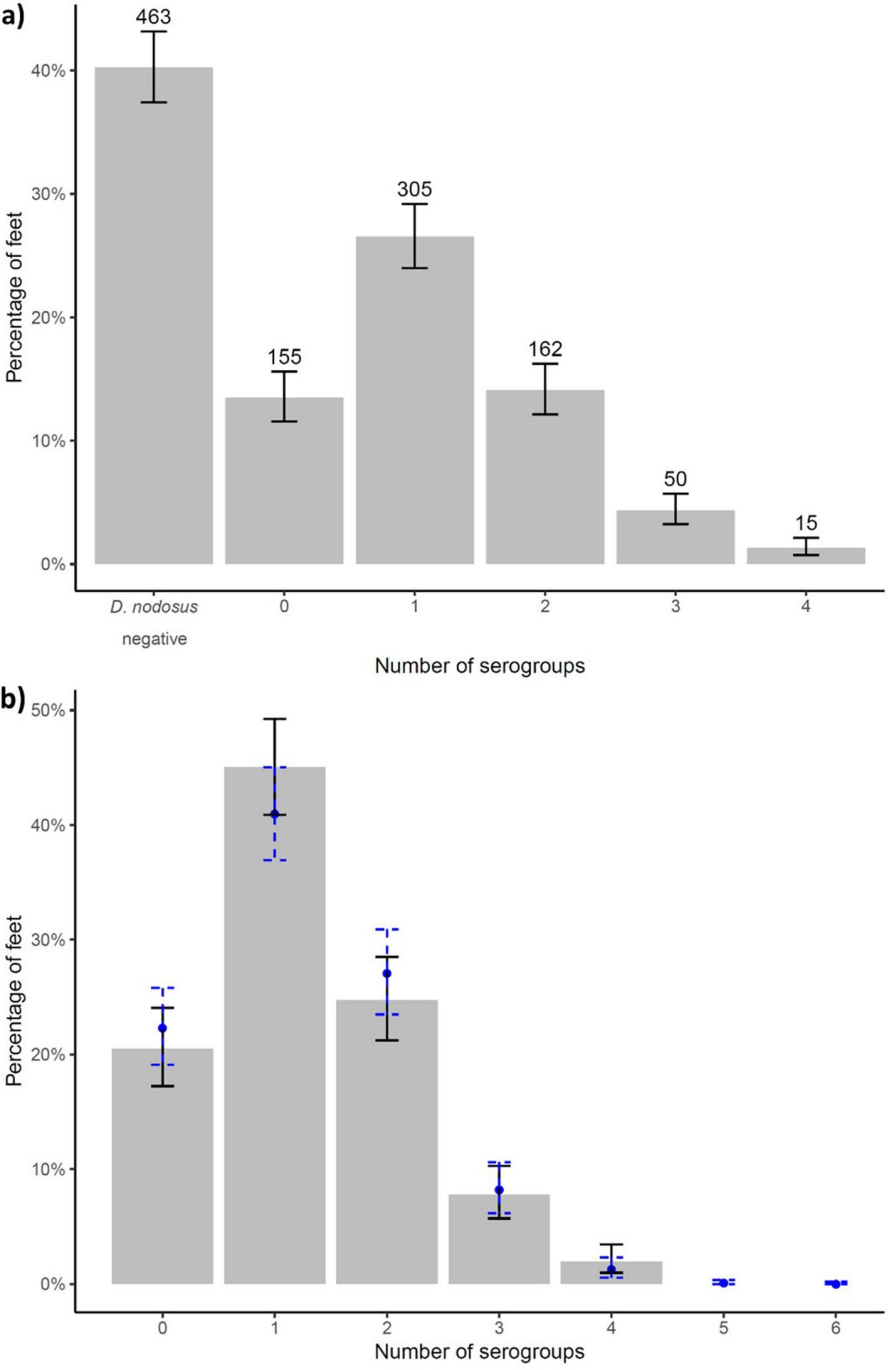
The number and percentage of feet by number of serogroups detected on feet with exact binomial 95% confidence intervals (solid error bars) from (**a**) all 1150 feet from 164 flocks and (**b**) from 566 *D. nodosus* positive footrot-affected feet from 138 flocks with the simulated expected percentage (point) and 95% distribution of the simulated data (dashed error bars) in blue.

**Figure 3:**
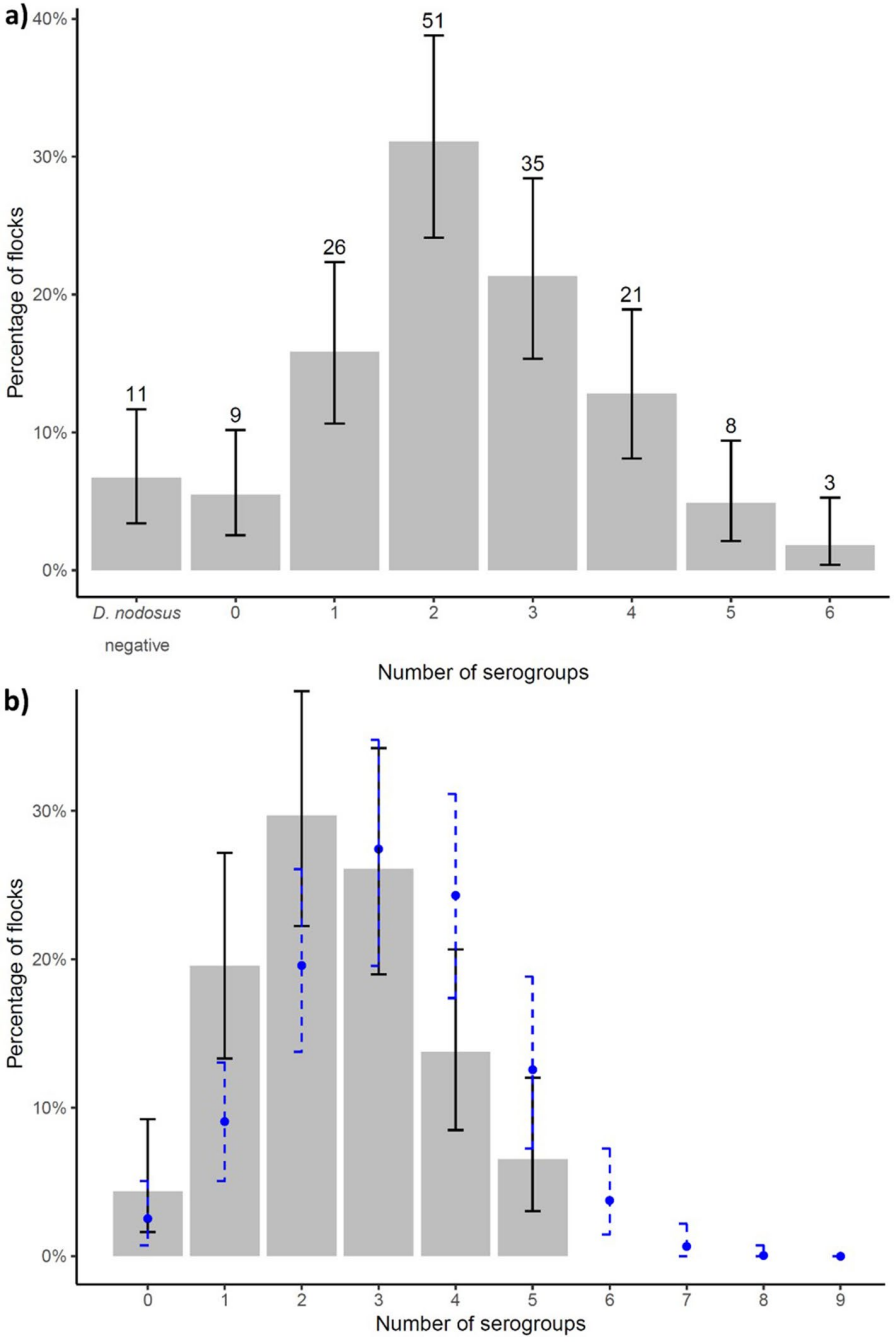
The number and percentage of flocks by number of serogroups detected with exact binomial 95% confidence intervals (solid error bars) from (**a**) all 1150 feet from 164 flocks and (**b**) from 566 *D. nodosus* and footrot positive feet from 138 flocks with the expected percentage (point) and 95% distribution of the simulated data (dashed error bars) in blue.

Not all *D. nodosus* positive samples were serogroup positive, swabs were more likely to be serogroup negative when the load of *D. nodosus* was low (Supplementary Fig. 1). The number of serogroups per foot and flock was skewed with a median of one (range 0–4) per foot (Fig. 2) and two (range 0–6) per flock (Fig. 3). There was no significant difference (Fisher’s exact test, *p* = 0.55) between the observed and expected number of serogroups per foot in Dataset III when data were simulated assuming no clustering of serogroups using the 566 *D. nodosus* positive, footrot-affected feet, indicating that the number of serogroups per foot was randomly distributed. However, again using simulated data, the distribution of number of serogroups per flock was significantly lower (Fisher’s exact test, *p* ≤ 0.01) than expected by chance in the 138 *D. nodosus* positive flocks, indicating that serogroups were clustered within flocks (Fig. 3b). There was evidence that the number of serogroups per flock was potentially higher than that detected, especially when there were fewer than three *D. nodosus* positive swabs from a flock (Table 2).

**Table 2:** Multivariable multinomial model of the number of serogroups detected from 153 flocks explained by number of swabs analysed and biosecurity factors.

Variable	Number of serogroups detected	N	%	OR	95% CI	*p* value
Number of *D. nodosus* positive swabs	≥ 3	67	43.8			
	**1**–**2**	77	50.3	**0.61**	**0.49**–**0.77**	**< 0.001**
	**0**	9	5.9	**0.19**	**0.09**–**0.43**	**< 0.001**
**Stocking rate (ref ≤ 4 ewes/acre)**						
< 4 ewes/acre	≥ 3	20	13.1			
	1–2	41	26.8			
	0	4	2.6			
≥ 4 ewes/acre	≥ 3	43	28.1			
	**1**–**2**	33	21.6	**0.40**	**0.19**–**0.85**	**0.017**
	0	5	3.3	0.81	0.15–4.34	0.805
No response	≥ 3	4	2.6			
	1–2	3	2.0	0.27	0.05–1.41	0.121

Terms where *p* < 0.05 are in bold. AIC = 231.17.

N = number of flocks, % = percentage of flocks, OR = odds ratio, 95% CI = 95% confidence interval.

### Prevalence and diversity of serogroups on feet and in flocks

**Figure 4:**
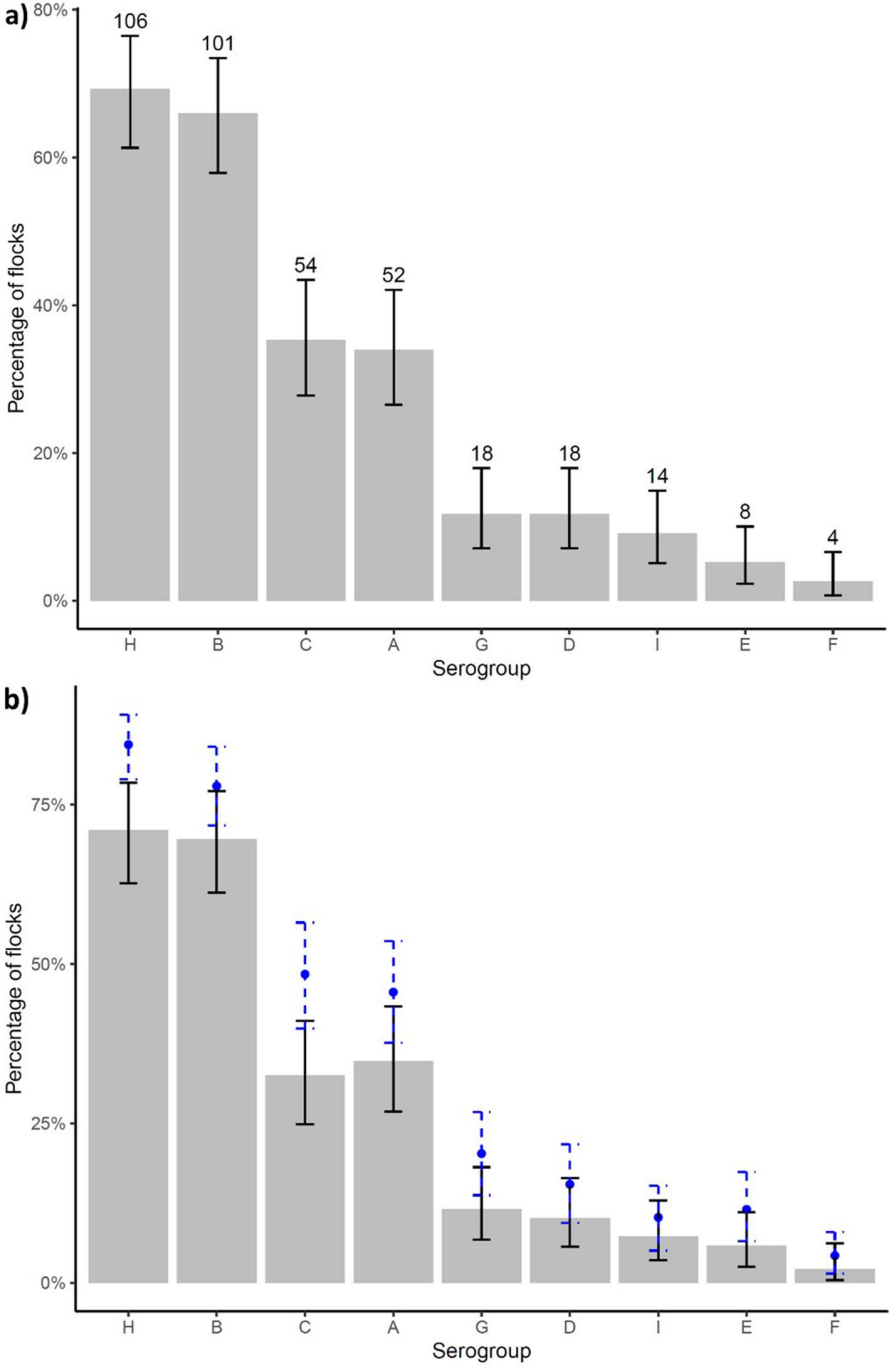
The number and percentage of flocks positive for each serogroup of *D. nodosus* with exact binomial 95% confidence intervals (solid error bars) for (**a**) 687 *D. nodosus* positive feet from 153 flocks and (**b**) 566 *D. nodosus* and footrot positive feet from 138 flocks with expected (point) and 95% distribution of the simulated data (dashed error bars) in blue.

**Figure 5:**
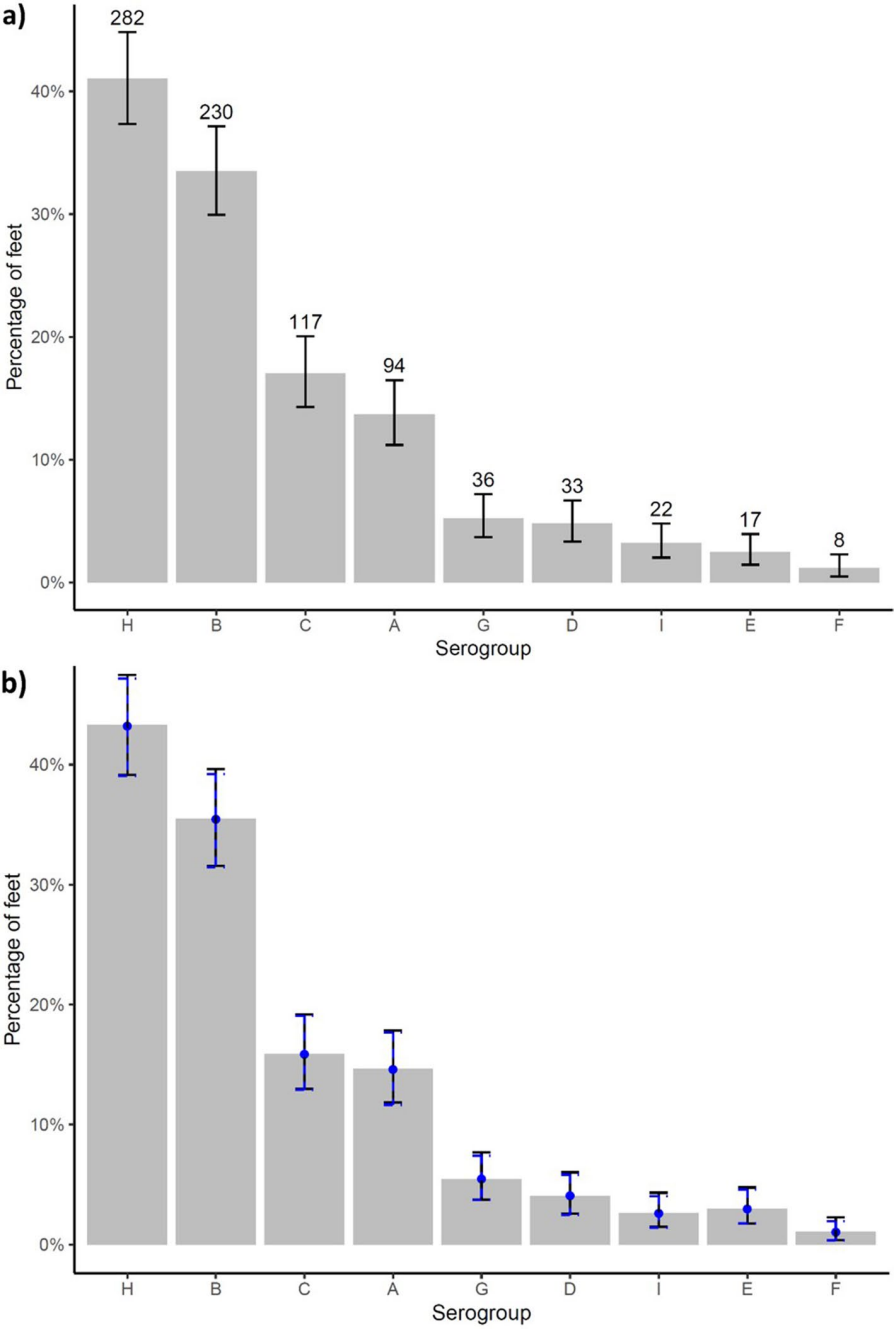
The number and percentage of feet positive for each serogroup of *D.* nodosus with exact binomial 95% confidence intervals (solid error bars) for (**a**) 687 *D. nodosus* positive feet from 153 flocks and (**b**) 566 *D. nodosus* and footrot-affected feet from 138 flocks with the expected (point) and 95% distribution of the simulated data (dashed error bars) in blue.

The prevalence of each serogroup varied from 2.6 to 69.3% per flock. Exact 95% confidence intervals around the point estimates indicate that serogroups H and B were distinctly the most commonly detected serogroups in > 60% of flocks (Fig. 4) and > 30% of feet (Fig. 5). This was followed by A and C and then serogroups D, E, F, G and I were detected at a similar prevalence in < 20% flocks and < 10% feet. The distribution of serogroups in flocks and on feet were similar, although there were fewer feet positive for all serogroups (Fig. 4 compared with Fig. 5) indicating that no serogroup was present in many flocks but on few feet, or vice versa. As with the number of serogroups, fewer flocks were positive for each serogroup than expected by chance (Fig. 4) indicating that serogroups were clustered within flocks. This is biologically plausible given that *D. nodosus* is infectious and so likely to amplify within flocks, but it might be explained in part by the limit of detection of a serogroup which was influenced by the test sensitivity^31^ and the number of *D. nodosus* positive swabs per flock (Table 2).

### Communities of serogroups in flocks

There were 50 combinations of serogroups across flocks. Using Raup-Crick analysis, only 7 pairs of flocks had communities of serogroups that were more different than would be expected by chance (β_RC_ > 0.95), and there were no pairs that were more similar than would be expected by chance (β_RC_ < − 0.95), out of 10,296 pairwise comparisons. The mean β_RC_ for all pairwise comparisons was − 0.16 (range − 0.93 to 0.99), therefore community assembly was highly stochastic. There were no regional clusters in a principal coordinates analysis of the data (Supplementary Fig. 2). Therefore, the communities of serogroups within flocks is random, with no geographical clustering.

### Multinomial model of disease state, number of swabs and biosecurity on the number of serogroups detected in flocks

A multivariable multinomial model was used to investigate factors associated with the number of serogroups detected in the 153 *D. nodosus* positive flocks (Dataset I). As the number of *D. nodosus* positive swabs per flock increased ≥ 3 serogroups were more likely to be detected than 1–2 or 0 serogroups (*p* < 0.01) and flocks with a stocking density of ≥ 4 ewes/acre compared with < 4 ewes/acre were more likely to have ≥ 3 serogroups than 1–2 serogroups (*p* = 0.017; Table 2). No other biosecurity variable was correlated with stocking density.

### Probability of detection of a serogroup in a flock

When serogroups were investigated from swab data from 270 sheep from 11 flocks in a clinical trial^32^, when there were 16–60 swabs per flock compared with up to 8 as in Dataset I, a mean of one extra serogroup was detected (Table 3). In total, 1–3 more serogroups were identified per flock from 8 (72.7%) flocks, 1 fewer serogroup was identified in 1 (9.1%) flock, and identical serogroups were detected in 3 (27.3%) flocks (Table 3). This confirms the finding that there was probably under detection of serogroups in some flocks in the current study.

**Table 3:** Serogroups detected in 11 flocks in the clinical trial^32^ from 7–8 single swabs (current study) and pooled samples with 16–60 swabs per sample.

Flock ID	Number of pooled samples	Number of swabs (current study)	Number of *D. nodosus* positive swabs (current study)	Serogroups detected
1	16	8	2	C
2	20	7	6	BCE*hi*
3	25	7	3	*a*B**c**H
4	29	7	4	*a*H
5	34	7	4	F
6	36	8	4	B*de*H*i*
7	54	8	6	ABH*i*
8	55	7	6	AB*c*EH
9	55	8	5	A*c*H
10	60	7	6	ABH
11	60	8	8	*a*BH

Serogroups detected from both studies are upper case, from pooled swabs are lower case italics, and from the current study are lower case underlined and bold.

The number of swabs analysed per flock to be 95% confident of detecting a serogroup that was truly present was calculated using the observed prevalence of each serogroup. There was a 95% likelihood of detecting serogroups that were present in 32% and 53% of samples with eight and four *D. nodosus* positive swabs (the median number of *D. nodosus* positive swabs per flock in this study) respectively (Supplementary Table 2). Therefore, serogroups with < 53% prevalence would not always have been detected.

### Theoretical protection offered by bivalent vaccines in England

Our results indicate that approximately 27% of flocks would be fully protected and 94% of flocks would be partially protected by a bivalent vaccine containing serogroups H and B (where full protection was because only serogroups H and B were present in the flock and partial protection was because other serogroups were in the flock). A total of 16 bivalent vaccines (out of the 36 possible combinations) against the two most common serogroups in all flocks would have fully protected 3–27% of flocks in the study and partially protected 39–94%. Sequential application of a further 2, 3, 4 and 5 bivalent vaccines in descending order of national serogroup prevalence (i.e. HB + CA + GD + IE + F) would have fully protected 65%, 83%, 97% and 100% of flocks, and partially protected 98%, 98%, 99% and 100% of flocks.

## Discussion

This is the largest and most representative study of the national prevalence and diversity of serogroups of *D. nodosus* in England to date. Key findings are that serogroups A–I are present and that the prevalence of serogroups varies. Serogroups are randomly distributed across England, clustered within flocks but not geographically co-located, with 50 combinations of serogroups across flocks, therefore one bivalent vaccine would not control footrot nationally.

The national prevalence of each serogroup varied considerably (Fig. 5) with three distinct prevalence groups, there is 95% probability of a true difference in the prevalence of B and H, A and C and then the remaining less prevalent serogroups. Serogroups H and B have been reported as the two most prevalent serogroups in the UK previously as far back as 30 years^11,14^, so the prevalence of each serogroup appears reasonably stable nationally over time. The high prevalence of serogroup B is also consistent with other countries where it is often the most prevalent serogroup^33–38^, however, serogroup H is not reported to be the most common serogroup in any other country. Despite the high prevalence of serogroups H and B a bivalent vaccine would protect only 27% of flocks in England and we conclude that one bivalent vaccine would not be effective in protecting the national flock.

The strength of the current study is the number of flocks investigated nationally which provides the robust estimates of the prevalence of serogroups. The percentage of flocks and feet with multiple serogroups and the maximum number of serogroups per flock are higher than reported in previous UK studies^12–14,39^ this is probably partly due to the increased sensitivity of PCR directly from DNA without culture^15^. Despite this, the number of serogroups detected in flocks is likely to be underestimated. Hill et al.^40^ calculated that over 40 swabs were required for a 95% chance of identifying all serogroups in Australian flocks, however this is likely to be lower with the smaller size of flocks in England, and with the increased sensitivity of the methods used here. If the aim is eradication of footrot by flock-specific vaccination, more intensive strategies are required to ensure that no serogroups are omitted. The farmers were not requested to ensure that the sheep that they took swabs from were representative of the flock, however flocks are typically kept in one group in the UK so it is expected that most samples were representative of the flock. Figure 3 indicates a slight left shift in distribution of observed than expected number of serogroups and that flocks would have approximately one more serogroup than observed. This was probably because some flocks submitted < 4 *D. nodosus* positive swabs which was associated with fewer serogroups detected (Table 2). The number of serogroups per swab might also be underestimated because of the limit of detection of the PCR test and because there is no specific PCR primer for serogroup M so it was not possible to estimate the prevalence of M. There is no reason to expect serogroup M to behave differently from other serogroups and so we would expect it to be randomly distributed across England, clustered within flocks and to increase the number of serogroup combinations further.

Few studies have investigated geographical distribution of serogroups in England^11,13^ and there is little published evidence of geographical co-location of serogroups in other countries, except possibly in India^41^, where breeds and managements are very different by region and fewer serogroups have been detected than in England and there is some evidence that the prevalence of serogroups is geographically clustered. In the current study, between flock serogroup communities were highly stochastic, with 50 combinations of serogroups detected in flocks and no clusters of flocks with more similar communities of serogroups, even in flocks within the same region (Supplementary Fig. 2) no two flocks had communities of serogroups that were more similar than would be expected by chance. Therefore, no bivalent candidate vaccines could protect flocks grouped geographically.

Lack of geographical clustering of serogroups indicates that serogroups of *D. nodosus* are randomly distributed across England. Footrot has been reported in the UK for over 200 years^42^ and over 90% of flocks have footrot^4^. *D. nodosus* persists on feet^6^ and so it seems likely that movement of infected sheep and poor biosecurity between flocks has contributed to its highly endemic profile. There is evidence for this in the current study where most flocks were open, 83.5% of the 164 farmers purchased sheep, often from distant locations^30^ and only 50% quarantined sheep for at least 3 weeks^30^, and less than 25% treated sheep with footrot or interdigital dermatitis on arrival^4^. It is also possible that within flock seroconversion of a serogroup occurred, this has been demonstrated in the laboratory^43^ and possibly in a UK flock^39^. Although the continuing dominance of serogroups H and B indicate national stability, the current study was limited to a single timepoint on each farm and so we could not determine stability of serogroups within flocks over time. Some longitudinal studies have reported that new serogroups ‘appear’ in flocks, although it is not possible to determine whether this is due to limits of detection or targeted vaccination altering the dominance of serogroups within a flock, or seroconversion^23,39^.

Flocks that were stocked at a density of ≥ 4 ewes/acre were more likely to have ≥ 3 serogroups detected than the 1–2 serogroups detected in less densely stocked flocks. This is a novel finding. This could be a true difference in number of serogroups in those flocks or it might be explained by increased transmission of *D. nodosus* between sheep within a densely stocked flock that increases the prevalence of rarer serogroups within a flock and therefore that they were more likely to be detected in the current study. Vaccination with Footvax, the nine-serogroup commercial vaccine, and biosecurity managements were not associated with number of serogroups or specific serogroups.

We conclude that the lack of dominance of the same two serogroups in flocks nationally or regionally make one national or a few regional bivalent vaccines unfeasible. With 50 communities of serogroups across 164 flocks we conclude that flock-specific bivalent vaccines would be essential.

## Materials and methods

This project was carried out according to approval from The University of Warwick Biomedical and Scientific Research Ethics Committee (REGO-2016-1758 AMO1) and Animal Welfare and Ethical Review Body (AWERB.24/15-16). Approval was granted on 23/02/16 and 08/04/16 respectively. All experiments were performed in accordance with relevant guidelines and regulations.

### Selection of farmers to the study

An invitation letter and reply slip were posted to 722 farmers in England in February and April 2016. These farmers had completed detailed questionnaires on lameness in their flock in 2013^4^, and also, for the majority, in 2014^44^, and indicated that they were willing to participate in further research. Replies were received between 09/02/2016–30/06/2016 from 192/722 (26.6%) farmers who agreed to participate. An additional 18 farmers who had participated in the original study^4^ and were participating in a separate clinical trial^32^ were recruited. There was no difference in the proportion of *D. nodosus* positive swabs or the number of serogroups detected from the swabs from the surveyed farms and the clinical trial (Table 4), so data were combined and are presented together.

**Table 4:** The number and percentage of swabs by disease state by 146 postal flocks and 18 clinical trial flocks^32^.

Foot disease status	All swabs (post)		All swabs (clinical trial)		All swabs		Number of serogroups	
	N	%	N	%	N	%	Median^a^	Range
Footrot	740	72.6	121	92.4	861	74.9	1	0–4
Healthy	78	7.7	0	0.0	78	6.8	1	0–3
Other	125	12.3	10	7.6	135	11.7	1	0–4
Unknown	76	7.5	0	0.0	76	6.6	1	0–4
Total	1019		131		1150			

60% of postal swabs were *D. nodosus* positive and 58% of clinical trial swabs.

Footrot = severe footrot or interdigital dermatitis lesion, Healthy = no lesion, Other = one or more lesions that did not include footrot, Unknown = lesion status not known.

^a^There was no difference in the number of serogroups detected between swabs from the post and the clinical trial (Fisher’s exact test, *p* = 0.613).

### Swab sample and questionnaire collection

Farmers participating by post were sent eight sterile swabs with charcoal amies gel transport medium (Thermo Scientific) and illustrated instructions on how to swab the feet^31^. Participants were asked to swab the interdigital skin of one foot of each of eight sheep ideally lame with footrot, that had not received treatment in the previous 2 weeks and to complete the sampling with healthy sheep if eight lame sheep were not present. They were not given any further instructions on which sheep to swab. Farmers recorded all lesions on the swabbed foot. Swabs were posted immediately at ambient temperature or were refrigerated overnight and arrived within 24 h of posting. On arrival, swabs, together with the amies medium, were immediately transferred to − 20 °C storage. Swabs were received between 02/03/16 and 08/09/16 from 146 farmers. The farmers who returned swabs also returned a completed three-page questionnaire regarding their flock in 2015. The 2015 questionnaire included questions on the presence and prevalence of four foot lesions, treatment and control managements for footrot, biosecurity practices, and some information about the flock in 2015.

The 18 flocks in the clinical trial had swabs from the interdigital skin of up to 15 lame sheep collected between November 2015 and August 2016. Eight swabs per flock were selected for the current project using the same criteria as for the farmers contacted by post. Sheep were selected in the following order of preference: lame with severe footrot or interdigital dermatitis (locomotion score ≥ 2^45^), lame with another lesion (locomotion score ≥ 2), not lame (locomotion score < 2). Feet were selected in the following order of preference: severe footrot lesion, interdigital dermatitis lesion, another lesion, no lesion. Swabs were stored in phosphate buffered saline. The liquid was spun off in a centrifuge and split into two aliquots, with one aliquot used for this project. The responses to the questionnaire for the farmers participating in the clinical trial^32^ were retrieved from a more detailed questionnaire conducted as part of that study.

### DNA extraction from swabs

Swabs were randomised for processing by assigning a random number generated in Microsoft Excel 2016 stratified by flock. DNA extraction was performed using a Nucleospin^®^ Tissue kit (Macherey–Nagel) with modifications. Swabs were thawed at room temperature, transferred to microcentrifuge tubes and lysis buffer T1 (400 μl) and proteinase K (40 μl) were added. Samples were vortexed for 5 s and incubated for 10 min at 56 °C. Post-incubation, lysis buffer B3 (400 μl) was added. Each sample was vortexed for 5 s and incubated at 70 °C for 5 min. Samples were cooled at room temperature for 5 min before being centrifuged at 12,000*g* for 1 min. The supernatant was added to 100% ethanol (400 μl) and centrifuged at 11,000*g* for 1 min and then loaded onto spin columns and centrifuged at 11,500*g* for 1 min. Flow-through was discarded and BW wash buffer (500 μl) was added to the spin columns and centrifuged at 11,000*g* for 1 min. Flow-through was discarded and B5 wash buffer (600 μl) was added to the spin columns and centrifuged at 11,000*g* for 1 min. Flow-through was discarded and spin columns were centrifuged for 1 min at 11,000*g* to dry the membrane. Spin columns were placed in microcentrifuge tubes and BE elution buffer (45 μl) heated to 70 °C was added and allowed to stand for 2 min. Tubes were spun at 11,000*g* for 1 min to elute DNA and extracted DNA was stored at − 20 °C.

### Screening for *Dichelobacter nodosus* with qPCR

To identify which samples contained *D. nodosus* and could therefore be tested for presence of each serogroup, each DNA sample was tested in triplicate using a qPCR that detected the *aprV2*/*aprB2* gene using the method published by Frosth et al.^46^ with some modifications. qPCR tests were used because they are more sensitive than the PCR tests. Modifications were made to the probe labelling (*aprV2*probe 6FAM-BHQ1, *aprB2*probe TxRd-BHQ2), Klearkall master mix (LGC Group) was used and only 1 μl of template DNA was used. The initial denaturation step was extended to 15 min as required by the master mix. *D. nodosus* strain VCS1703A was the positive control for *aprV2*, and strain C305 was the positive control for *aprB2* (Supplementary Table 3). Each qPCR plate contained a no template control in triplicate to differentiate noise from true positives and in addition, only samples that were 3/3 positive for either *aprV2* or *aprB2* were classed as positive which was a conservative way of defining positive samples and limiting the risk of false positive results. Total *D. nodosus* load in a sample was calculated as the sum of the number of genome copies of *aprB2* and *aprV2*. Most samples that were positive for *D. nodosus* in only 1 or 2 of the 3 replicate qPCR tests had low loads.

### Detection of serogroups from DNA using PCR

Samples positive for *D. nodosus* by qPCR were tested separately for nine serogroups (A–I) of *D. nodosus* using single serogroup PCRs. Serogroup M was not investigated because there is no published PCR test. Multiplex serogroups PCRs were tested but these were less sensitive^31^. The primers and program were published by Dhungyel et al.^47^. Each reaction (25 μl) contained MyTaq Red Mix (Bioline) (12.5 μl), forward primer (0.5 μM), reverse primer (0.5 μM) and bovine serum albumin (0.5 mg/ml). Products were electrophoresed on 3% agarose gels stained with ethidium bromide and visualised under ultraviolet light. Positive controls were normalised to 10 ng/μl and are in Supplementary Table 3.

### Data entry and analysis

Data was entered manually into Microsoft Excel and rechecked once to ensure accuracy. All cases where farmers did not answer the question were coded as No Response. There was no difference in the number of serogroups detected per swab between the post and clinical trial farmers (Table 4). Data analysis was conducted in R statistical software (version 3.5.1)^48^. The significance level used for all tests was 0.05. Kruskal–Wallis tests were used to investigate whether flock size, the prevalence of lameness or the prevalence of severe footrot in ewes, were associated with the number of swabs submitted and the number of swabs that were *D. nodosus* positive per flock because this data was not normally distributed.

### Detection of serogroups of *Dichelobacter nodosus* by swab and flock

Three datasets were created:

(I) 1150 swabs from 164 flocks.
(II) 566 *D. nodosus* positive swabs from footrot-affected feet from 138 flocks.
(III) A randomly simulated dataset of Dataset II.

For simulated Dataset III, the proportion of swabs positive for each serogroup in Dataset II was calculated. Each swab was then assigned as positive or negative for each serogroup (A–I) weighted by the observed proportion of swabs positive. Only swabs from footrot-affected feet were simulated to remove the heterogeneity in probability of detection of *D. nodosus* from other lesions, and because the majority of *D. nodosus* positive swabs were from footrot-affected feet (Table 1). The simulation from the observed data was run 1000 times assuming a random distribution of serogroups present within and between flocks. Differences in the number of serogroups in feet and flocks between the observed and simulated data were tested with Fisher’s exact tests. Post hoc analysis of chi-square and Fisher’s exact tests were conducted with the fifer (version 1.1)^49^ and rcompanion (version 2.1.1)^50^ packages respectively.

### Multinomial model of disease state, number of swabs and biosecurity managements on the number of serogroups detected

Unordered multinomial logistic regression models^51^ with ≥ 3, 1–2, or 0 serogroups per flock were used to investigate whether the number of swabs submitted/analysed and biosecurity managements in the flock were associated with the number of serogroups detected per flock. The nnet package (version 7.3-12)^52^ was used. The univariable model results of variables considered for inclusion in the model are in Supplementary Tables 4 and 5. The models were built using a manual forward stepwise procedure by sequentially testing each term using variables with the lowest Akaike’s Information Criterion (AIC) at each iteration. Variables were retained in the final multivariable models if *p* ≤ 0.05 and > 1 flock was in each significantly different category (Supplementary Table 5). All remaining variables were retested in the final model^53^. Chi-square tests and Fisher’s exact tests were used to test for correlations between the biosecurity terms in the final model and all other tested biosecurity terms. The models took the form:

$$logit({\pi }_{1k}/{p}_{i0k}) ={\beta }_{0k} +\Sigma {\beta }_{0}X +{e}_{k}$$

$$logit({\pi }_{2k}/{p}_{i0k}) ={\beta }_{1k} +\Sigma {\beta }_{1}X +{e}_{k}$$

where $logit({\pi }_{1k}/{p}_{i0k})$ is the probability of having 1–2 serogroups detected versus ≥ 3 and $logit({\pi }_{2k}/{p}_{i0k})$ is the probability of having 0 serogroups detected versus ≥ 3, ${\beta }_{0k}$ and ${\beta }_{1k}$ are constants for 1–2 serogroups and 0 serogroups, ${\beta }_{0}X$ and ${\beta }_{1}X$ are coefficients for number of swabs and biosecurity variables X for 1–2 serogroups and 0 serogroups, and ${e}_{k}$ is the residual random error that follows a binomial distribution.

### Spatial analysis of the diversity of serogroups of *Dichelobacter nodosus* in England

Raup-Crick analysis of community diversity was conducted using null models following the method described by Chase et al.^54^. Principal coordinates analysis^55^ of the Raup–Crick analysis was used to visually assess for geographical clusters using the ape package (version 5.2)^56^.

### Probability of detection of a serogroup from the swabs

In the current study there were up to eight swabs per flock. For 11 of the clinical trial flocks, > 8 swabs (range 16–60) were pooled and the number of serogroups per pooled sample was analysed in a separate study (Monaghan et al., unpublished data). For these flocks each foot of 15 sheep was swabbed and pooled into one of ten footrot disease severity states (Supplementary Material 1). The presence of each serogroup was investigated using the same PCR method as used in this study. The number of serogroups detected per flock when eight (the current study) and more than eight swabs (clinical trial) were collected were calculated to investigate whether the number of serogroups detected per flock was associated with the number of *D. nodosus* positive swabs analysed.

### Prevalence and probability of detection of a serogroup

In order to calculate the probability of detecting common and rare serogroups in a flock, the minimum numbers of swabs required to have a 95% probability of detection of serogroups by prevalence X was calculated using the formula:

$$Prevalence \,X= 1 - \sqrt[n]{0.05}$$

where n is the number of swabs.

## Supplementary information

Supplementary file1.

## Data Availability

The datasets supporting the conclusions of this study are available on request.
