# Supplementary material for: Serogroups of Dichelobacter nodosus, the cause of footrot in sheep, are randomly distributed across England
Source: Sci Rep. 2020 Oct 8;10:16823. doi: 10.1038/s41598-020-73750-5 (PMC7546612; doi:10.1038/s41598-020-73750-5)
Supplement: Supplementary file 1 — Supplementary file1. [file 41598_2020_73750_MOESM1_ESM.docx]

**Serogroups of *Dichelobacter nodosus,* the cause of footrot in sheep, are randomly distributed across England**

Naomi S Prosser, Emma M Monaghan, Laura E Green, Kevin J Purdy

**Supplementary Table 1:** Kruskal-Wallis tests for associations with the number of swabs submitted per 164 English sheep flocks.

| Factor | Test statistic | p value |
| --- | --- | --- |
| All swabs |  |  |
| Prevalence of lameness in ewes in 2015 | 4.87 | 0.301 |
| Prevalence of severe footrot in ewes in 2015 | 2.54 | 0.637 |
| Ewe flock size in 2015 | 4.96 | 0.292 |
| *D. nodosus* positive swabs |  |  |
| Prevalence of lameness in ewes in 2015 | 7.55 | 0.479 |
| Prevalence of severe footrot in ewes in 2015 | 3.56 | 0.895 |
| Ewe flock size in 2015 | 10.43 | 0.236 |

**Supplementary Table 2:** The minimum prevalence of a serogroup on swabs for a 95% likelihood of detection of the serogroup from 1 – 299 swabs.

| Number of swabs | Prevalence |
| --- | --- |
| 1 | 0.95 |
| 2 | 0.78 |
| 3 | 0.64 |
| 4 | 0.53 |
| 5 | 0.46 |
| 6 | 0.40 |
| 7 | 0.35 |
| 8 | 0.32 |
| 9 | 0.29 |
| 10 | 0.26 |
| 11 | 0.24 |
| 12 | 0.23 |
| 13 | 0.21 |
| 14 | 0.20 |
| 15 | 0.19 |
| 16 | 0.18 |
| 17 | 0.17 |
| 18 | 0.16 |
| 19 | 0.15 |
| 20 | 0.14 |
| 22 | 0.13 |
| 24 | 0.12 |
| 26 | 0.11 |
| 29 | 0.10 |
| 32 | 0.09 |
| 36 | 0.08 |
| 42 | 0.07 |
| 49 | 0.06 |
| 59 | 0.05 |
| 74 | 0.04 |
| 99 | 0.03 |
| 149 | 0.02 |
| 299 | 0.01 |

**Supplementary Table 3:** The strain and source of the positive controls used in the *D. nodosus* serogroup specific PCRs.

| *D. nodosus* strain | Serogroup positive control | Location of isolation | Source |
| --- | --- | --- | --- |
| V2_3LR | A | University of Warwick | Giebel^1^ |
| 12 | B | University of Warwick | Muzafar et al.^2^ |
| C305 | C | University of Sydney | Claire Russell, Bristol University |
| 20 | D | University of Warwick | Muzafar et al.^2^ |
| E | E | Unknown | Claire Russell, Bristol University |
| JIR_3568 | F | Charles Sturt University | Ruth Kennan, Monash University |
| VCS1703A | G | University of Sydney | Julian Rood, Monash University |
| 0107 -EDH | H | University of Warwick | Smith et al.^3^ |
| 0108 -EDI | I | University of Warwick | Smith et al.^3^ |

**Supplementary** **Table 4:** Univariable multinomial models of the number of serogroups per flock, 153 flocks with *D. nodosus* positive swabs, by the number of swabs submitted, positive. Terms where p < 0.05 are in bold.

| Number of serogroups (ref = ≥ 3) | OR | 95% CI | p value | AIC |
| --- | --- | --- | --- | --- |
| Swabs submitted |  |  |  | 271.89 |
| 1 – 2 | 0.41 | 0.11 – 1.48 | 0.174 |  |
| 0 | 0.36 | 0.08 – 1.60 | 0.180 |  |
| **Swabs submitted from footrot-affected feet** |  |  |  | **268.77** |
| **1 – 2** | **0.82** | **0.70 – 0.97** | **0.020** |  |
| 0 | 0.79 | 0.58 – 1.06 | 0.112 |  |
| Contaminated swabs |  |  |  | 275.24 |
| 1 – 2 | 1.04 | 0.65 – 1.66 | 0.868 |  |
| 0 | 0.86 | 0.31 – 2.36 | 0.769 |  |
| ***D. nodosus* positive swabs** |  |  |  | **230.83** |
| **1 – 2** | **0.61** | **0.49 – 0.77** | **< 0.001** |  |
| **0** | **0.20** | **0.09 – 0.43** | **< 0.001** |  |
| ***D. nodosus* positive swabs from footrot lesions** |  |  |  | **243.44** |
| **1 – 2** | **0.68** | **0.57 – 0.82** | **< 0.001** |  |
| **0** | **0.38** | **0.23 – 0.62** | **< 0.001** |  |
| *D. nodosus* positive swabs from healthy feet |  |  |  | 273.13 |
| 1 – 2 | 1.38 | 0.64 – 2.94 | 0.408 |  |
| 0 | < 0.01 | < 0.01 – 3.65e29 | 0.865 |  |
| *D. nodosus* positive swabs from non-footrot lesions | |  |  | 274.62 |
| 1 – 2 | 0.87 | 0.56 – 1.34 | 0.518 |  |
| 0 | 1.18 | 0.55 – 2.52 | 0.676 |  |
| *D. nodosus* positive swabs from unknown lesion |  |  |  | 271.84 |
| 1 – 2 | 1.16 | 0.80 – 1.69 | 0.433 |  |
| 0 | < 0.01 | < 0.01 – 6.40e20 | 0.809 |  |
| OR = odds ratio, CI = confidence interval, AIC = Akaike’s Information Criterion | | | | |

**Supplementary** **Table 5**: Univariable multinomial model results of the number of serogroups detected from *D. nodosus* positive swabs from footrot-affected feet in 153 flocks explained by biosecurity factors. Terms where p < 0.05 are in bold.

| Variable | Number of serogroups (ref = ≥ 3) | Number of flocks | OR | 95% CI | AIC / p value |
| --- | --- | --- | --- | --- | --- |
| Used routine footbathing to manage footrot | | | | | 273.99 |
| No | ≥ 3 | 27 |  |  |  |
|  | 1 – 2 | 26 |  |  |  |
|  | 0 | 5 |  |  |  |
| Yes | ≥ 3 | 38 |  |  |  |
|  | 1 – 2 | 51 | 1.39 | 0.70 – 2.76 | 0.341 |
|  | 0 | 4 | 0.57 | 0.40 – 2.32 | 0.430 |
| No response | ≥ 3 | 2 |  |  |  |
|  | 1 – 2 | 0 | - | - | - |
|  | 0 | 0 | - | - | - |
| Vaccinated to manage footrot | | | | | 272.48 |
| No | ≥ 3 | 46 |  |  |  |
|  | 1 – 2 | 43 |  |  |  |
|  | 0 | 5 |  |  |  |
| Yes | ≥ 3 | 19 |  |  |  |
|  | 1 – 2 | 34 | 1.91 | 0.95 – 3.85 | 0.068 |
|  | 0 | 4 | 1.94 | 0.47 – 8.01 | 0.361 |
| No response | ≥ 3 | 2 |  |  |  |
|  | 1 – 2 | 0 | - | - | - |
|  | 0 | 0 | - | - | - |
| Sheep vaccinated against footrot | | | | | 274.28 |
| None | ≥ 3 | 46 |  |  |  |
|  | 1 – 2 | 43 |  |  |  |
|  | 0 | 5 |  |  |  |
| Ewes | ≥ 3 | 14 |  |  |  |
|  | 1 – 2 | 26 | 1.99 | 0.92 – 4.30 | 0.081 |
|  | 0 | 4 | 2.63 | 0.62 – 11.14 | 0.190 |
| Not all ewes | ≥ 3 | 5 |  |  |  |
|  | 1 – 2 | 8 | 1.71 | 0.52 – 5.64 | 0.377 |
|  | 0 | 0 | - | - | - |
| No response | ≥ 3 | 2 |  |  |  |
|  | 1 – 2 | 0 | - | - | - |
|  | 0 | 0 | - | - | - |
| Frequency of Footvax^TM^ use | | | | | 274.06 |
| Never | ≥ 3 | 46 |  |  |  |
|  | 1 – 2 | 43 |  |  |  |
|  | 0 | 5 |  |  |  |
| Once/yr | ≥ 3 | 16 |  |  |  |
|  | 1 – 2 | 29 | 1.94 | 0.93 – 4.06 | 0.079 |
|  | 0 | 2 | 1.15 | 0.20 – 6.52 | 0.875 |
| >once/yr | ≥ 3 | 3 |  |  |  |
|  | 1 – 2 | 5 | 1.78 | 0.40 – 7.91 | 0.448 |
|  | 0 | 2 | 6.12 | 0.82 – 45.85 | 0.078 |
| No response | ≥ 3 | 2 |  |  |  |
|  | 1 – 2 | 0 | - | - | - |
|  | 0 | 0 | - | - | - |
| Started vaccination | | | | | 281.93 |
| Did not vaccinate | ≥ 3 | 46 |  |  |  |
|  | 1 – 2 | 43 |  |  |  |
|  | 0 | 5 |  |  |  |
| > 0 – 1 year | ≥ 3 | 4 |  |  |  |
|  | 1 – 2 | 8 | 2.14 | 0.60 – 7.62 | 0.241 |
|  | 0 | 0 | - | - | - |
| > 1 – 2 years | ≥ 3 | 3 |  |  |  |
|  | 1 – 2 | 7 | 2.50 | 0.61 – 10.28 | 0.205 |
|  | 0 | 2 | 6.13 | 0.82 – 45.93 | 0.077 |
| > 2 – 5 years | ≥ 3 | 6 |  |  |  |
|  | 1 – 2 | 5 | 0.89 | 0.25 – 3.14 | 0.858 |
|  | 0 | 0 | - | - | - |
| > 5 years | ≥ 3 | 4 |  |  |  |
|  | 1 – 2 | 9 | 2.41 | 0.69 – 8.39 | 0.168 |
|  | 0 | 1 | - | - | 0.492 |
| No response | ≥ 3 | 4 |  |  |  |
|  | 1 – 2 | 5 | 1.34 | 0.34 – 5.31 | 0.680 |
|  | 0 | 1 | - | - | - |
| Separated lame sheep to manage footrot | | | | | 272.71 |
| No | ≥ 3 | 45 |  |  |  |
|  | 1 – 2 | 42 |  |  |  |
|  | 0 | 5 |  |  |  |
| Yes | ≥ 3 | 20 |  |  |  |
|  | 1 – 2 | 35 | 1.91 | 0.94 – 3.90 | 0.075 |
|  | 0 | 4 | 1.08 | 0.18 – 6.37 | 0.936 |
| No response | ≥ 3 | 2 |  |  |  |
|  | 1 – 2 | 0 | - | - | - |
|  | 0 | 0 | - | - | - |
| **Culled sheep that had been lame** | | | | | 275.02 |
| After 1/2 bouts | ≥ 3 | 11 |  |  |  |
|  | 1 – 2 | 13 |  |  |  |
|  | 0 | 1 |  |  |  |
| **After 3/more bouts** | ≥ 3 | 2 |  |  |  |
|  | 1 – 2 | 7 | 2.96 | 0.51 – 17.26 | 0.228 |
|  | **0** | **3** | **16.51** | **1.09 – 250.32** | **0.043** |
| Persistently / severe disease / misshapen hoof | ≥ 3 | 43 |  |  |  |
|  | 1 – 2 | 41 | 0.81 | 0.32 – 2.00 | 0.644 |
|  | 0 | 5 | 1.28 | 0.14 – 12.12 | 0.829 |
| Never | ≥ 3 | 9 |  |  |  |
|  | 1 – 2 | 15 | 1.41 | 0.45 – 0.47 | 0.557 |
|  | 0 | 0 | - | - | - |
| No response | ≥ 3 | 2 |  |  |  |
|  | 1 – 2 | 1 | - | - | - |
|  | 0 | 0 | - | - | - |
| Flock mixed with other flocks | | | | | 274.05 |
| No | ≥ 3 | 63 |  |  |  |
|  | 1 – 2 | 68 |  |  |  |
|  | 0 | 9 |  |  |  |
| Yes | ≥ 3 | 2 |  |  |  |
|  | 1 – 2 | 1 | - | - | - |
|  | 0 | 0 | - | - | - |
| No response | ≥ 3 | 2 |  |  |  |
|  | 1 – 2 | 8 | 0.46 | 0.04 – 5.24 | 0.534 |
|  | 0 | 0 | - | - | - |
| Purchased sheep | | | | | 277.63 |
| No | ≥ 3 | 12 |  |  |  |
|  | 1 – 2 | 10 |  |  |  |
|  | 0 | 1 |  |  |  |
| Yes | ≥ 3 | 53 |  |  |  |
|  | 1 – 2 | 66 | 1.49 | 0.60 – 3.73 | 0.389 |
|  | 0 | 8 | 1.81 | 0.21 – 15.90 | 0.592 |
| No response | ≥ 3 | 2 |  |  |  |
|  | 1 – 2 | 1 | - | 0.05 – 7.63 | 0.694 |
|  | 0 | 0 | - | - | - |
| Purchased sheep from market | | | | | 277.95 |
| No | ≥ 3 | 27 |  |  |  |
|  | 1 – 2 | 30 |  |  |  |
|  | 0 | 5 |  |  |  |
| Yes | ≥ 3 | 38 |  |  |  |
|  | 1 – 2 | 44 | 1.04 | 0.53 – 2.05 | 0.905 |
|  | 0 | 4 | 0.57 | 0.14 – 2.31 | 0.430 |
| No response | ≥ 3 | 2 |  |  |  |
|  | 1 – 2 | 3 | 1.35 | 0.21 – 8.70 | 0.752 |
|  | 0 | 0 | - | - | - |
| Purchased sheep from private farm sale | | | | | 278.08 |
| No | ≥ 3 | 37 |  |  |  |
|  | 1 – 2 | 43 |  |  |  |
|  | 0 | 4 |  |  |  |
| Yes | ≥ 3 | 28 |  |  |  |
|  | 1 – 2 | 31 | 0.95 | 0.49 – 1.87 | 0.889 |
|  | 0 | 5 | 1.65 | 0.41 – 6.72 | 0.484 |
| No response | ≥ 3 | 2 |  |  |  |
|  | 1 – 2 | 3 | 1.29 | 0.20 – 8.14 | 0.786 |
|  | 0 | 0 | - | - | - |
| Frequency of sheep purchases in last 5yrs | | | | | 275.35 |
| ≤ once/yr | ≥ 3 | 30 |  |  |  |
|  | 1 – 2 | 45 |  |  |  |
|  | 0 | 4 |  |  |  |
| > once/yr | ≥ 3 | 23 |  |  |  |
|  | 1 – 2 | 18 | 0.52 | 0.24 – 1.13 | 0.098 |
|  | 0 | 4 | 1.30 | 0.29 – 5.78 | 0.726 |
| No response | ≥ 3 | 14 |  |  |  |
|  | 1 – 2 | 14 | 0.67 | 0.28 – 1.60 | 0.363 |
|  | 0 | 1 | - | - | - |
| Regions sheep purchased from | | | | | 285.10 |
| No purchase | ≥ 3 | 12 |  |  |  |
|  | 1 – 2 | 10 |  |  |  |
|  | 0 | 1 |  |  |  |
| Multiple regions | ≥ 3 | 7 |  |  |  |
|  | 1 – 2 | 7 | 1.20 | 0.31 – 4.59 | 0.790 |
|  | 0 | 3 | 5.14 | 0.44 – 59.47 | 0.190 |
| The North / Scotland | ≥ 3 | 15 |  |  |  |
|  | 1 – 2 | 16 | 1.28 | 0.43 – 3.83 | 0.659 |
|  | 0 | 1 | - | - | - |
| The Midlands / Wales | ≥ 3 | 8 |  |  |  |
|  | 1 – 2 | 20 | 3.00 | 0.93 – 9.70 | 0.066 |
|  | 0 | 2 | 3.00 | 0.23 – 38.88 | - |
| The South East / East Anglia | ≥ 3 | 6 |  |  |  |
|  | 1 – 2 | 11 | 2.20 | 0.60 – 8.08 | 0.235 |
|  | 0 | 1 | - | - | - |
| The South West | ≥ 3 | 16 |  |  |  |
|  | 1 – 2 | 9 | 0.67 | 0.21 – 2.18 | 0.511 |
|  | 0 | 1 | - | - | - |
| Don’t know | ≥ 3 | 0 |  |  |  |
|  | 1 – 2 | 1 | - | - | - |
|  | 0 | 0 | - | - | - |
| No response | ≥ 3 | 3 |  |  |  |
|  | 1 – 2 | 3 | 1.20 | 0.20 – 7.31 | 0.773 |
|  | 0 | 0 | - | - | - |
| Quarantined sheep arriving on farm for at least 3 weeks | | | | | 282.46 |
| Always | ≥ 3 | 29 |  |  |  |
|  | 1 – 2 | 38 |  |  |  |
|  | 0 | 3 |  |  |  |
| Sometimes | ≥ 3 | 14 |  |  |  |
|  | 1 – 2 | 17 | 0.93 | 0.39 – 2.18 | 0.862 |
|  | 0 | 2 | 1.38 | 0.21 – 9.23 | 0.739 |
| Never | ≥ 3 | 9 |  |  |  |
|  | 1 – 2 | 12 | 1.02 | 0.38 – 2.74 | 0.973 |
|  | 0 | 2 | 2.15 | 0.31 – 14.94 | 0.440 |
| N/A | ≥ 3 | 11 |  |  |  |
|  | 1 – 2 | 9 | 0.62 | 0.23 – 1.71 | 0.358 |
|  | 0 | 1 | - | - | - |
| No response | ≥ 3 | 4 |  |  |  |
|  | 1 – 2 | 1 | - | - | - |
|  | 0 | 1 | - | - | - |
| **Stocking rate** | | | | | 270.37 |
| < 4 ewes/acre | ≥ 3 | 20 |  |  |  |
|  | 1 – 2 | 41 |  |  |  |
|  | 0 | 4 |  |  |  |
| **≥ 4 ewes/acre** | ≥ 3 | 43 |  |  |  |
|  | **1 – 2** | **33** | **0.37** | **0.19 – 0.75** | **0.006** |
|  | 0 | 5 | 0.58 | 0.14 – 2.40 | 0.453 |
|  | ≥ 3 | 4 |  |  |  |
| No response | 1 – 2 | 3 | 0.37 | 0.07 – 1.79 | 0.215 |
|  | 0 | 0 | - | - | - |
| Flock shared grazing with cattle | | | | | 278.03 |
| No | ≥ 3 | 36 |  |  |  |
|  | 1 – 2 | 39 |  |  |  |
|  | 0 | 4 |  |  |  |
| Yes | ≥ 3 | 29 |  |  |  |
|  | 1 – 2 | 37 | 1.18 | 0.61 – 2.29 | 0.629 |
|  | 0 | 5 | 1.55 | 0.38 – 6.31 | 0.539 |
| No response | ≥ 3 | 2 |  |  |  |
|  | 1 – 2 | 1 | - | - | - |
|  | 0 | 0 | - | - | - |
| OR = odds ratio, CI = confidence interval, AIC = Akaike’s Information Criterion, - = no flocks in one of the comparison groups | | | | | |

**Supplementary Figure 1:** The load of genome copies of *D. nodosus* per μl for 687 *D. nodosus* positive swabs with 0 – 4 serogroups detected.


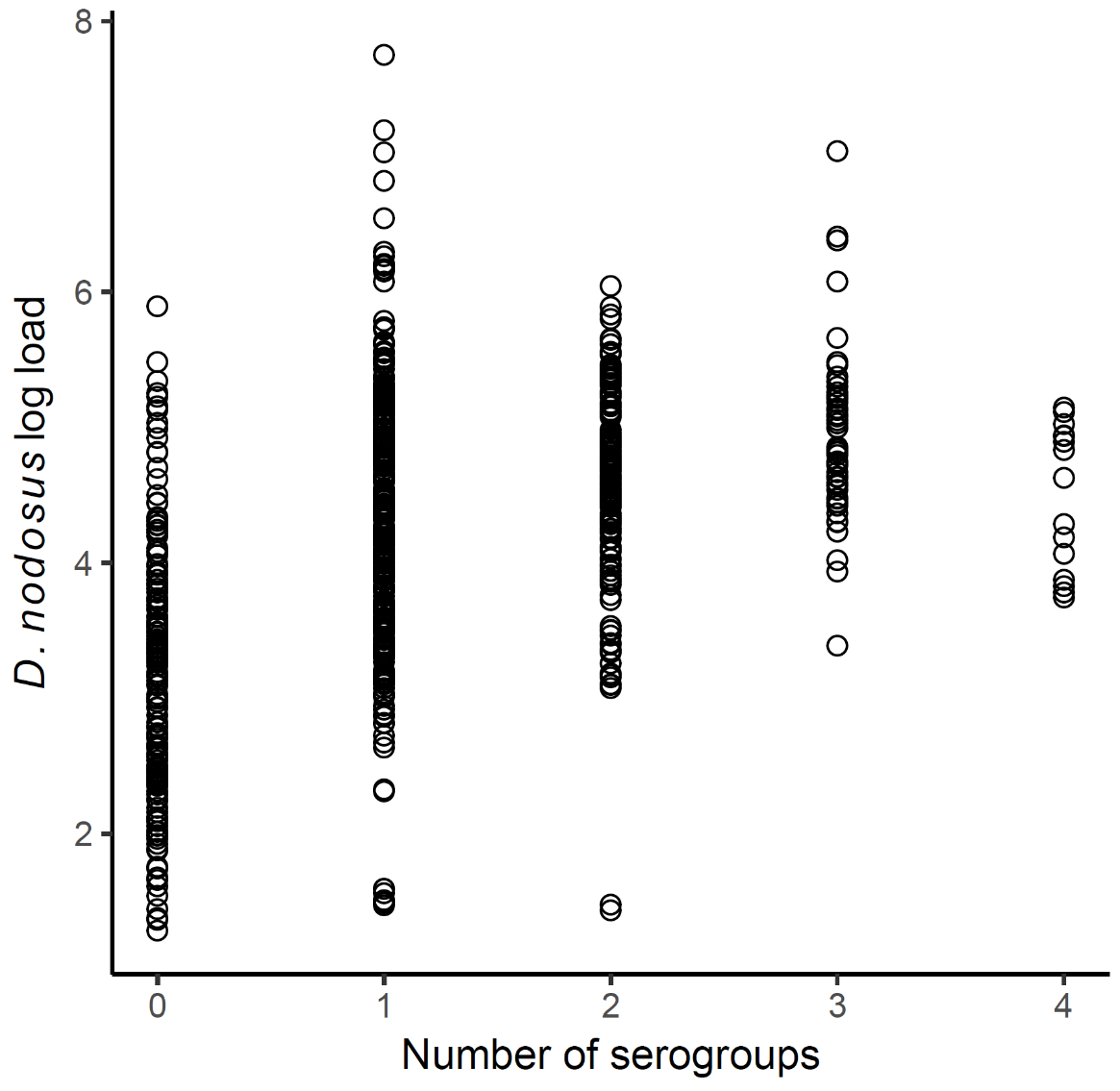


**Supplementary Figure 2:** The first two axes of a principal coordinates analysis of the β_RC_ distance metric for each of 144 flocks with ≥ 1 serogroup split into all regions.

**Supplementary Material 1:** Footrot lesion scoring system.

**Interdigital dermatitis (ID) lesion classification**

1. Clean interdigital foot with no dermatitis (scald) lesions.
2. Slight interdigital dermatitis, partial loss of hair, slight redness but dry.
3. Slight interdigital dermatitis, partial/complete loss of hair, redness, pasty scum (<10% of the interdigital area affected).
4. Moderate interdigital dermatitis, partial/complete loss of hair, redness, pasty scum (10-50% of the interdigital area affected).
5. Severe interdigital dermatitis, partial/complete loss of hair, redness, pasty scum (>50% affected).

**Footrot (SFR) lesion classification (damage to tissue at the worst affected point). Depth measurement as a comparison to the digit’s width at its mid-length point**

1. No under-running of the wall of the digit.
2. Depth of lesion less than 25% of the width of the digit’s sole at its mid-length point. Lesion relatively dry but foul smelling.
3. Depth of lesion at or more than 25% but less than 50% of the width of the digit’s sole at its mid-length point. Lesion wet and foul smelling.
4. Depth of lesion at or more than 50% but less than 75% of the width of the digit’s sole at its mid-length point. Lesion wet and foul smelling.
5. Depth of lesion 100% of the width of the digit’s sole at its mid-length point. Lesion wet and foul smelling.

**References**

1 Giebel, K. *Persistence of Dichelobacter nodosus, the causal agent of ovine footrot* PhD thesis, University of Warwick, (2017).

2 Muzafar, M. *et al.* The role of the environment in transmission of *Dichelobacter nodosus* between ewes and their lambs. *Vet. Microbiol.* **179**, 53-59, (2015).

3 Smith, E. M. *et al.* Within-flock population dynamics of *Dichelobacter nodosus*. *Front. in Vet. Sci.* **4**; 58; 10.3389/fvets.2017.00058 (2017).
